# Investigation of the possibility of fermentation of red grape juice and rice flour by *Lactobacillus* *plantarum* and *Lactobacillus* *casei*

**DOI:** 10.1002/fsn3.2461

**Published:** 2021-08-01

**Authors:** Royaossadat Mirmohammadi, Nafiseh Zamindar, Seyed Hadi Razavi, Mehrosadat Mirmohammadi, Saeed Paidari

**Affiliations:** ^1^ Department of Food Science and Technology Young Researchers Club Shahrekord Branch Islamic Azad University Shahrekord Iran; ^2^ Department of Food Science and Technology Isfahan (Khorasgan) Branch Islamic Azad University Isfahan Iran; ^3^ Department of Food Science and Engineering Faculty of Biosystem Engineering University of Tehran Tehran Iran; ^4^ Department of Chemistry Shahreza Branch Islamic Azad University Shahreza Iran

**Keywords:** grape juice, lactic acid fermentation, *Lactobacillus casei*, *Lactobacillus plantarum*, rice flour

## Abstract

The aim of the current study was to evaluate the possibility of the bacterial growth and substrate metabolism during the fermentation of red grape juice and the mixture of red grape juice and rice flour solution using *Lactobacillus plantarum* and *Lactobacillus casei*. In recent years, cereal‐based beverages have been used as functional compounds such as antioxidants, dietary fiber, minerals, probiotics, and vitamins in diets. In this research, fermentation of red grape juice (media 1) and 1:1 mixture of red grape juice and rice flour solution (media 2) by two strains of gram positive and homofermentative lactic acid bacteria: *L. plantarum* and *L*. *casei* (individually and mixed) was examined. Fermentation was carried out at 37°C for 48 hr. Microbial population, pH, acidity, sugar, and organic acid metabolism were measured during the fermentation period. Data showed that in media 2 fermented with mixed culture of both *L*. *plantarum* and *L*. *casei*, acidity and microbial population increased sharply at the initial stages of fermentation, and the most percentage of lactic acid production occurred. Red grape juice fermented with mixture of *L. plantarum* and *L. casei* showed the most sugar consumption (*p* < .05). Results indicated that the use of the mixture of red grape juice and rice flour solution can be a proper substrate for producing lactic acid.

## Graphical abstract

This paper mainly covers fermentation of red grape and rice and evaluating chemical content of produced material.

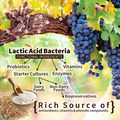

## 1 INTRODUCTION

Microorganisms and their fermentative activities have had a distinctive role in human being as old as civilization (Erten et al., 2014; Mith et al., 2014). A broad variety of foods and beverages have been produced by fermentative activities of microorganisms, which finally led to desirable biochemical changes in different foods (Blandino et al., 2003; Ferreira et al., 2000). Decreasing the volume of material to be transported, increasing the nutritive value, degrading undesirable component, reducing the cooking time, energy consumption, and making safer products are other benefits of fermentation activities (Hosseini et al., 2017; Simango, 1997). Lactose intolerance and high cholesterol level of dairy products are the main drawbacks of fermented dairy products and the consumer demand for nondairy functional foods and beverages (Adejuwon et al., 2020; Prado et al., 2008).

Due to the need to implement a sustainable food system, devastating impact of the dairy industry on the environment, the water needed to run a dairy farm, negative impacts on the environment and climate changes, production of large amount of waste, consumption of antibiotics and chemicals, and soil contamination, it is expected to control the consumption of dairy products. Fruit juice and fermented cereals can be utilized as a substitute for nondairy beverages with a new field of research dealing with the health promoting features of so called "functional foods" (Gobbetti et al., 2010; Nkhata et al., 2018). Grape juice is physicochemically similar to grapes except a difference within the value of crude fiber and the seed oil (Haight & GuMP, 1995; Mildner‐Szkudlarz et al., 2013). Next to sugars, the organic acids found in grapes are the second largest group of compounds accounting for nearly 1% of solids present in grape juice (Mendes Ferreira & Mendes‐Faia, 2020). Di cagno and Servi (Abedi & Sahari, 2014; Di Cagno et al., 2010), used concentrated red grape as substrate for making a functional fermented beverages having selected lactic acid bacteria regarded as the most important bacteria concerning food fermentation, pharmaceutical, and special dietary applications. Similar studies have been performed on the production of nondairy fermented foods and beverages with cereals and fruits (Mousavian et al., 2021). The distinguishing feature on this research is the combination of rice flour with grape juice and simultaneous fermentation with *L. plantarum* and *L. casei*. (Coda et al., 2012; Haghighatpanah et al., 2020; Haight & GuMP, 1995; Jiang et al., 2010; Liu et al., 2014).

Moreover, lactic acid fermented fruits and fruit juice are common in some Middle Eastern countries. Lactic acid fermented grape juice, fermented cherry, and Medlar can be mentioned as example of these beverages (Arici & Coskun, 2001; Do Quynh Nguyen et al., 2021; Paidari & Ibrahim, 2021).

Rice (*Oryza sativa* L.) is a staple food for over half of the world's population. It is considered as one of the significantly gluten‐free vital food crops all over the world and being a unique crop due to its white color, soft taste, low sodium levels, easily digestible carbohydrates, and hypoallergenic properties. Despite its numerous advantages, rice proteins have poor functional properties. One of the main byproducts of rice processing is broken rice. Broken rice is separated from head rice during rice milling process and is known to occur to approximately 1%–13% of milled rice(Ahari et al., 2016; Arora & Padua, 2010). Most broken rice is mainly used as animal feed and rice flour for pastry. Therefore, rice flour is an attractive raw material for manufacturing foods and beverages and fermentation with lactic acid bacteria (LAB) may improve its quality as a gluten‐free product (Chiş et al., 2020; Nafiseh et al., 2021).

Cereal based beverages can satisfy the market demand for nondairy beverages and also may provide functional compounds such as antioxidants, dietary fiber, minerals, probiotics, and vitamins (Coda et al., 2011; Ding & Li, 2021; Paidari & Ahari, 2021).

Grape juice concentrate is produced from *vitis vinifera* grapes in Iran and rice flour is a byproduct of rice processing which is considered as a kind of waste. The aim of this research was to investigate the growth rate and substrate metabolism during the fermentation of red grape juice and the mixture of red grape juice and rice flour solution (1:1) by single and mixed selected lactic acid bacteria. The fermented product may provide some functional properties in a nondairy beverage for sustainable food system and protecting the environment.

## 2 MATERIALS AND METHODS

### 2.1 Strains and cultures

Probiotic lactic acid bacteria *Lactobacillus plantarum* subsp. *plantarum* (DSMZ 20174), *Lactobacillus* casei subsp. Casei (DSMZ 20011) were supplied by the (GmbH). Bacterial cultures were stored at −70°C in MRS medium (Merck) containing 40% of glycerol. The strains were reactivated by means of double passage on MRS (Ghorbani et al., 2021; Jahandideh et al., 2012; Mousavi et al., 2013; Yoon et al., 2005). Commercial concentrated red grape juice was supplied from Takdaneh Iran Co. and kept at −20°C prior to use.

### 2.2 Fermentation process

Lactic acid bacteria (*L. plantarum* and *L. casei*) were cultivated until the late exponential phase of growth was reached (ca. 9h), centrifuged at 4,500 rpm for 10 min and the biomass was introduced into the all media (Coda et al., 2011; Mousavi et al., 2011). Treatments were divided in two different media. The first media including pasteurized red grape juice (Brix=20˚) inoculated with *Lactobacillus plantarum* (GLp), *Lactobacillus casei* (GLc) and 1:1 ratio of *Lactobacillus plantarum* and *Lactobacillus casei* (GLpLc), the second including mixture of red grape juice (Brix=20˚) and rice flour solution 6% (which was separately pasteurized) were inoculated with *Lactobacillus plantarum* (GRLp), *Lactobacillus casei* (GRLc) and 1:1 ratio of *Lactobacillus plantarum* and *Lactobacillus casei* (GRLpLc). All media were incubated at 37°C for 48 hr and samples were taken every 12 hr for chemical and microbiological analysis (Wu et al., 2021).

### 2.3 Microbiological analysis

Viable cell were determined by the standard plate count method using MRS medium, Serial dilutions (with 0.9% NaCl solution) of treatments were prepared. Aliquots of 1 ml of dilution were plated in MRS agar plates, then the plates were incubated at 37°C for 48 hr. (Wu et al., 2021). And the microbial population was expressed as colony‐forming units per milliliter (c.f.u/ml) (Jahandideh et al., 2012; Mousavi et al., 2011; Mousavi et al., 2013; Paidari et al., 2021; Yoon et al., 2005).

### 2.4 Chemical analysis

Chemical changes were determined by sampling during the fermentation at 12 hr intervals. A digital pH meter (Metrohm 827) was used for pH measurements. Total acidity, expressed as tartaric acid percent, was determined by titrating by 0.1 N NaOH (Merck) to pH 8.1 (Jahandideh et al., 2012; Mousavi et al., 2011). Quantitative analysis of organic acids (lactic and citric) were carried out by HPLC (Agilent) apparatus equipped with a UV detector. A separation column (zorbax EBC C18) set at 25°C by 0.01% phosphoric acid as the mobile phase and injection volume of 20 μl was used at a flowrate of 1 ml/min. Organic acids content were reported using external standards(Jahandideh et al., 2012). Sugars (fructose and glucose) were measured by HPLC (Knauer) equipped with a Knauer Analytisch refractive index (RI) detector. A separation column (Eurokat Ca 300 × 4 mm) was employed and distilled water was used as mobile phase. The flow rate of mobile phase was 1 ml/min and the operation temperature was 70°C. The volume of the injected sample for each run was 20 μl. Sugar content was reported using external standards (Crha & Pazourek, 2020).

### 2.5 Statistical analysis

Since the initial inoculation amounts for each treatment was not exactly equal, analysis of covariance was performed using SAS (9.1 software Institute Inc.). The analysis indicated that the effect of the initial inoculation amount was not significant (*p* < .05); therefore, the analysis of variance were performed. The experiment was conducted in a factorial form, using a completely randomized design to study the effects of time, media, and microorganism on microbial growth, pH, acidity, sugars, and organic acids. Media in two levels (red grape juice and 1:1 mixture of red grape juice and rice flour solution), lactic acid bacteria in three levels (*L. plantarum*, *L. casei* and 1:1 ratio of *L. plantarum* and *L. casei*) and time in four levels (12, 24, 36, and 48 hr) were independent variables. Experiments were carried out in triplicate for pH, acidity, and microbial population and duplicate for organic acids and sugars. Mean comparison was carried out using LSD test (*p* < .05). The results were expressed as mean ± *SD* (standard deviation).

## 3 RESULTS AND DISCUSSION

The effect of bacteria, media, time, and their intraction on pH, acidity, microbial population, glucose, fructose, and organic acids were presented in Tables 1 and 2.

**TABLE 1:** The effect of bacteria, media, time and their interactions on pH, acidity and microbial population

	Degree of freedom	Mean square		
		pH	Acidity	cfu/ml
Bacteria	2	0.014**	0.031**	3.73E + 17*
Media	1	0.353**	0.113**	2.76E + 18**
Time	4	0.934**	0.414**	1.99E + 18**
Bacteria*Media	2	0.01**	0.008*	6.34E + 16^ns^
Bacteria*Time	8	0.004**	0**	6.73E + 17**
Media*Time	4	0.202**	0.058**	1.89E + 17^ns^
Bacteria*Media*Time	8	0.002**	0.001**	2.84E + 17**

Abbreviation: ns, not significant.

* Significantly different (*p* < .05).

** Significantly different (*p* < .01).

**TABLE 2:** The effect of bacteria, media, time and their interactions on glucose, fructose and organic acids

	Degree of freedom	Mean square			
		Glucose	Fructose	Lactic acid	Citric acid
Bacteria	2	1.463^ns^	0.273^ns^	928,321.369**	100,239.265^ns^
Media	1	120.817^ns^	196.607*	72,516,467.550**	132,907.634^ns^
Time	2	55.441^ns^	48.332^ns^	465,885,655.003**	314,142.565**
Bacteria*Media	2	0.978^ns^	3.786^ns^	6,840,550.415**	99,455.204^ns^
Bacteria*Time	4	16.691^ns^	13.105^ns^	2,218,826.265**	17,840.646^ns^
Media*Time	2	18.49^ns^	27.349^ns^	18,290,218.291**	23,601.864^ns^
Bacteria*Media*Time	4	18.619^ns^	21.826^ns^	2,516,907.932**	88,212.999^ns^

Abbreviation: ns, not significant.

* Significantly different (*p* < .05).

** Significantly different (*p* < .01).

### 3.1 Growth kinetics

**FIGURE 1:**
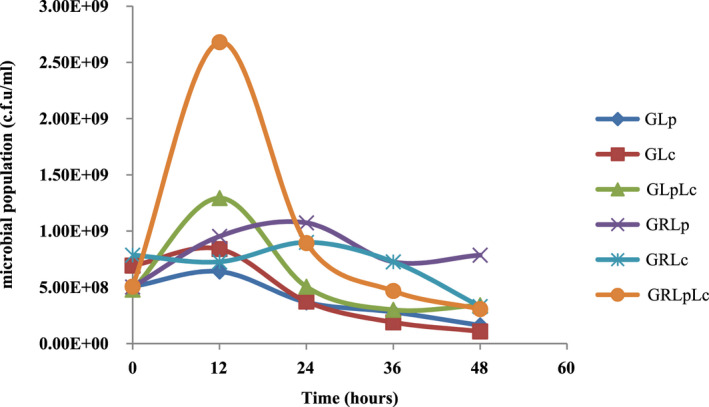
Kinetics of fermentation process. (GLp = fermentation of grape juice) by *L. plantarum*, GLc = fermentation of grape juice by *L. casei*, GLpLc = fermentation of grape juice by 1:1 mixture of *L. plantarum* and *L. casei*, GRLp = fermentation of (1:1) mixture of grape juice and rice flour solution by *L. plantarum*, GRLc = fermentation of (1:1) mixture of grape juice and rice flour solution by *L. casei*, GRLpLc = fermentation of (1:1) mixture of grape juice and rice flour solution by (1:1) mixture of *L. plantarum* and *L. casei*

The kinetics of fermentation process for each treatment was presented in Figure 1. The highest microbial population was observed within 12 hr in GLp, GLc, GLpLc, and GRLpLc and within 24 hr in GRLp and GRLc. Three‐way interaction of bacteria, media, and time on microbial population was significant (*p* < .05). Mean comparisons of three‐way interaction findings showed that the highest microbial population was obtained by GRLpLc and GLpLc, respectively, within 12 hr of fermentation, indicating that the mixture of both bacteria caused a faster growth rate in both media. However, when rice flour is added to the grape juice (media 2), a significant increasing in microbial population was observed (*p* < .05).

In the mixture of red grape juice and rice flour solution *L. plantarum* showed a faster growth rate comparing to *L. casei*. Similar results were reported by Mousavi et al. (Mousavi et al., 2011). Mousavi reported higher growth rate for *L. plantarum* in pomegranate juice compared to *L. paracasei*. These results are contrary to the results obtained by Jahandideh et al. (Jahandideh et al., 2012). They studied fermentation of Echium extract by the same lactic acid bacteria. According to the results, it is assumed that the growth of different bacteria in various media depends firmly on the initial pH of the extract. In grape and pomegranate juice having high acidic pH, *L. plantarum* could grow better and *L. paracasei* had lower growth rate but in *Echium* extract with an initial pH of 6.5 *L. paracasei* exhibited highest growth comparing to other strains. In other studies by Silveira et al. (Silveira et al., 2012) and kim et al. (Kim et al., 2012), the suitability of, respectively, cashew apple juice and potato juice (with high initial pH) as substrate for lactic acid fermentation was investigated. They expressed that cashew apple juice and potato juice could be suitable substrate for the growth of *L. casei* and lactic acid production without the need of B vitamins and/or yeast extract addition. Silveira et al. (Silveira et al., 2012) reported that the effect of initial pH and temperature on microbial growth and viability depends on the substrate and the strain under study. Figure 1 showed that the microbial population decreased with a sharp slope after log phase in most of treatments, which might be due to the fermentation temperature (37°C) in present study. The fermentation temperature in similar studies was 30°C (Mousavi et al., 2011; Mousavi et al., 2013). Yáñez et al. (Yáñez et al., 2008) also reported that low pH of medium can lead to the decrease in the maximum growth rate and an extended length of the lag phase.

### 3.2 pH and acidity

**FIGURE 2:**
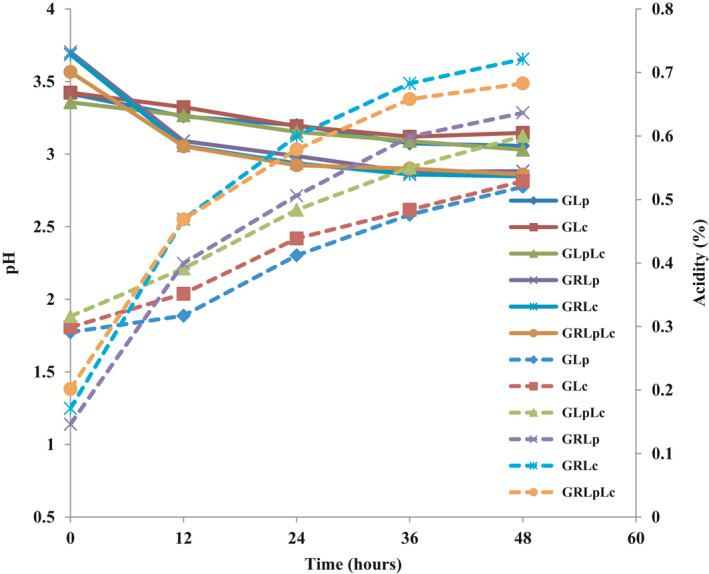
pH and titrable acidity changes during fermentation. (♦ = fermentation of grape juice) by *L. plantarum* (GLp),■ = fermentation of grape juice by *L. casei* (GLc), ▲ = fermentation of grape juice by 1:1 mixture of *L. plantarum* and *L. casei* (GLpLc), × = fermentation of 1:1 mixture of grape juice and rice flour solution by *L. plantarum* (GRLp), * = fermentation of 1:1 mixture of grape juice and rice flour solution by *L. casei* (GRLc), ● = fermentation of 1:1 mixture of grape juice and rice flour solution by 1:1 mixture of *L. plantarum* and *L. casei* (GRLpLc). Dash lines indicate acidity changes during fermentation)

The pH changes indicate a sharp slope reduction within first 12 hr of fermentation following by slow trend of decrease in all media. In the red grape juice, pH decreased slowly during the fermentation (Figure 2). Total acidity of both substrates increased following the same trend (Figure 2). Acid tolerance is an important probiotic characteristic for surviving during fermentation in food medium and results of this study are in agreement with other researchers (Holzapfel & Schillinger, 2002; Mousavi et al., 2013).

### 3.3 Sugar consumption during the fermentation process

**FIGURE 3:**
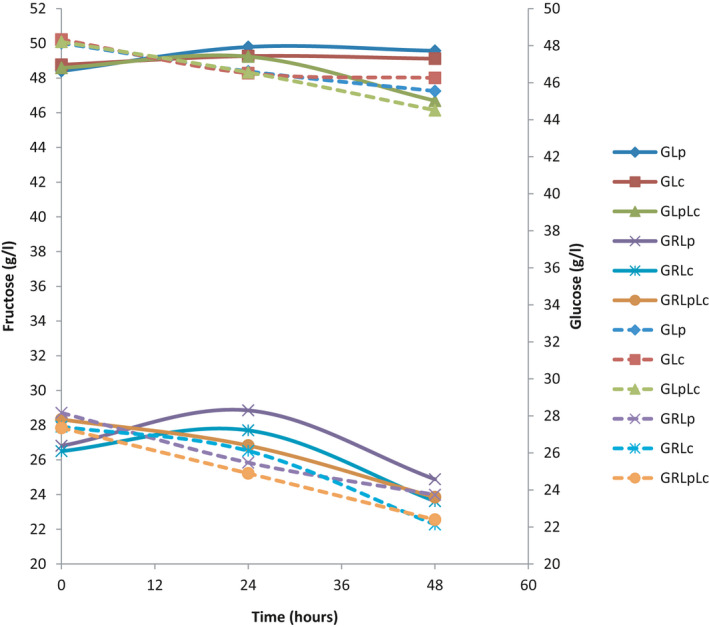
Glucose and fructose changes during fermentation.. (♦ = fermentation of grape juice) by *L. plantarum* (GLp), ■ = fermentation of grape juice by *L. casei* (GLc), ▲ = fermentation of grape juice by 1:1 mixture of *L. plantarum* and *L. casei* (GLpLc), × = fermentation of 1:1mixture of grape juice and rice flour solution by *L. plantarum* (GRLp), * = fermentation of 1:1 mixture of grape juice and rice flour solution by *L. casei* (GRLc), ● = fermentation of 1:1 mixture of grape juice and rice flour solution by 1:1 mixture of *L. plantarum* and *L. casei* (GRLpLc). Dash lines indicate glucose changes during fermentation

Since, half of media 2 was composed of grape juice, the initial value of glucose and fructose in medium 2 was half of medium 1. Figure 3 shows sugar analysis results obtained by HPLC. Comparing glucose and fructose consumption, all bacterial strains metabolized glucose before fructose. Wang et al. (Wang et al., 2003) also reported that glucose is a superior carbon and energy source for lactobacilli and bifidobacteria. For both glucose and fructose in media 1 and 2, the mixture of *L. plantarum* and *L. casei* consumed more sugars than individual cultures. In medium 1, *L. plantarum* showed more affinity to sugar consumption compared to *L. casei*, which is in agreement with the results obtained by Yoon et al. (Yoon et al., 2006) on the fermentation of cabbage juice by some strains of probiotic lactic acid bacteria. Mousavi et al. (Mousavi et al., 2013) found that in fermentation of pomegranate juice *L. plantarum* showed more affinity to sugar consumption followed by *L. delbrueckii*, *L. acidophilus,* and *L. paracasei,* respectively.

### 3.4 Citric acid consumption

Citrate metabolism is considered an unstable trait in lactic acid bacteria. This instability is due to location of the citrate permease gene on a plasmid. The initial breakdown of citrate and the conversion of the intermediate pyruvate into specific fermentation products can be regulated on different levels, depending on the microorganism. The pyruvate, which is formed from citrate breakdown will be converted by one or more of the pyruvate‐utilizing enzymes depending on the cultivation conditions (Hugenholtz, 1993). The key enzymes of citrate metabolism are: (a) citrate permease, an enzyme with a crucial role in the uptake of citrate into the cell, which its activity is strongly dependent on the pH; (b) citrate lyase enzyme, through which citrate is split into acetate and oxaloacetate (OAA); and (c) OAA‐decarboxylase, which decarboxylates OAA to pyruvate in *oenococcus oeni*, as it has been found in *Lactococcus spp*. (Mendes Ferreira & Mendes‐Faia, 2020).

**FIGURE 4:**
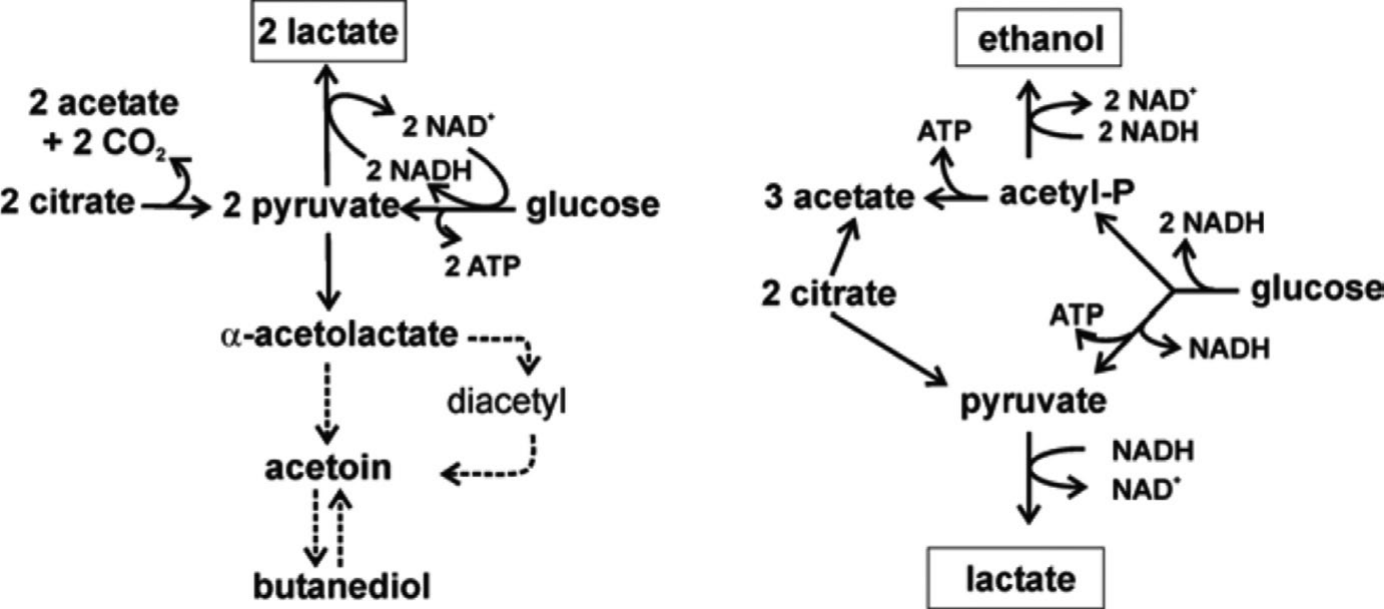
Co‐metabolism of citrate and glucose in LAB. Left, homofermentative bacteria. Right, heterofermentative bacteria

Bacteria from the genera *Lactococcus* and *Enterococcus* as well as *L. plantarum* degrade sugars (such us glucose or lactose) by a homofermentative pathway forming lactic acid as the main end product (Figure 4). During the cometabolism of one of these sugars with citrate, large amounts of pyruvate are generated, which is converted to the production of the C_4_ aroma compounds.

In the presence of glucose and citrate, each mol of citrate produces one mol of pyruvate without generating NADH. This produces an intracellular excess of pyruvate that is diverted to the synthesis of α‐acetolactate and subsequently to the production of the C_4_ aroma compounds. The end‐products of this cometabolism are lactate diacetyl, acetoin γ‐butanediol (García‐Quintáns et al., 2008).

**FIGURE 5:**
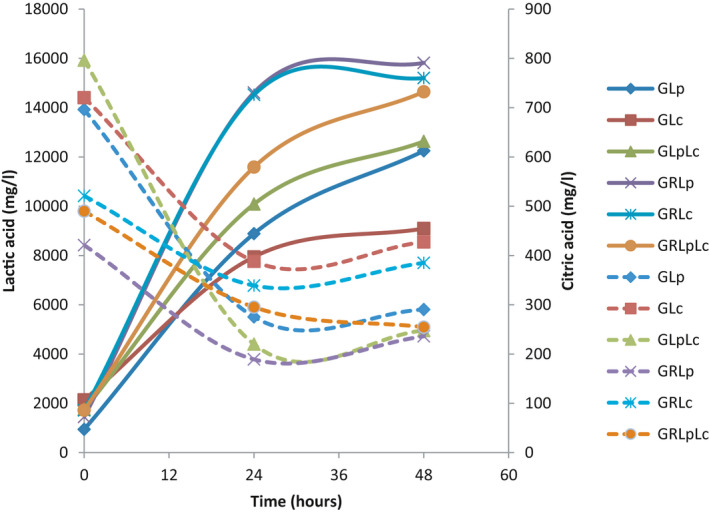
Lactic and citric acid changes during fermentation.. (♦ = fermentation of grape juice) by *L. plantarum* (GLp),■ = fermentation of grape juice by *L. casei* (GLc), ▲ = fermentation of grape juice by 1:1 mixture of *L. plantarum* and *L. casei* (GLpLc), × = fermentation of 1:1 mixture of grape juice and rice flour solution by *L. plantarum* (GRLp), * = fermentation of 1:1 mixture of grape juice and rice flour solution by *L. casei* (GRLc), ● = fermentation of 1:1 mixture of grape juice and rice flour solution by 1:1 mixture of *L. plantarum* and *L. casei* (GRLpLc). Dash lines indicate citric acid changes during fermentation

In this study, probiotic lactic acid bacteria were capable of metabolizing citric acid as a carbon source during the fermentation. Citric acid reduced more quickly during initial 24 hr of fermentation in all treatments (Figure 5). This process resulted in reduction of citric acid in all treatments. As you can see in the Figure 4, for glucose to be turned into pyruvate, the recovery of NADH is needed. Also, for this recovery, excess amount of lactate in the media is needed. However, pyruvate synthesis from citrate is not dependent on NAD^+^. In the beginning of fermentation, there is not enough lactic acid present in the media (Figure 4). Citrate is synthesized faster and after 24 hr, when the lactic acid concentration is at highest level, citrate consumption almost stops. However, as Figure 3 showed, the amount of glucose reduced with a slight slope from the beginning of fermentation with a rise in lactate.

*Lactobacillus plantarum* and *Lactobacillus casei* and GLp showed the most citric acid consumption (*p* < .05), respectively. In a similar study, the selected probiotic bacteria were capable of metabolizing citric acid in pomegranate juice soon after fermentation started. Researchers reported there was very low sugar content in the pomegranate juice and in contrast considerable amounts of citric acid and the strains metabolized citric acid as the major carbon source available in pomegranate juice (Mousavi et al., 2013). In the present study, inspite of high amount of initial sugars in grape juice, the selected lactic acid bacteria consumed more amount of citric acid in comparison with sugars. Other researchers reported that metabolism of carbohydrates by *Lactobacillus* varies from strain to strain and depends on the substrate and even on the fermentation time (Hou et al., 2000).

As could be seen in Table 2, the amount of citric acid was affected by time and the other parameter had not significant effect on citric acid (*p* < .01). Figure 5 illustrated that after 24 hr citric acid decrease stopped due to lactate production and initiating glucose conversion to pyruvate.

### 3.5 Lactic acid production

**FIGURE 6:**
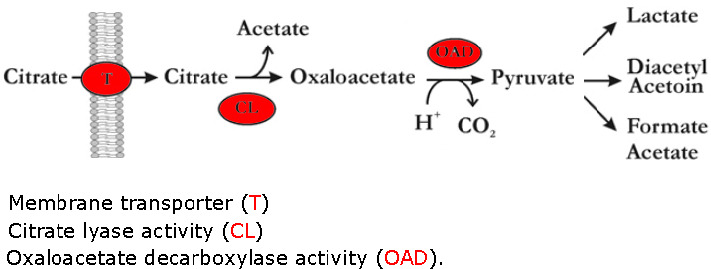
Consumption of citrate in bacterial cells and its by products

Since lactic acid is recognized as the main metabolite produced by lactic acid bacteria (De Vries & Stouthamer, 1967), the kinetics of the production of lactic acid during the fermentation was studied (Figure 5). Also fermentation process of citrate is illustrated in Figure 6. Results indicated that lactic acid was produced by all the strains and its concentration increased as the fermentation commenced. Fermentation of medium 2 by *L. plantarum* and *L. casei* individually, within 48 hr of the fermentation, produced the most amount of lactic acid (although, the major level of this amount was observed within initial 24 hr of fermentation). The lowest production of lactic acid was observed in fermentation of medium 1 by *L. casei*. Accordingly, *L. plantarum* showed more capabilities to produce lactic acid comparing to *L. casei* and the mixture of both. Fermentation of medium 2 by *L. plantarum* after 48 and 24 hr, produced the most amount of lactic acid, respectively. Some authors also reported that the concentration of lactic acid increased very quickly during fermentation (Coda et al., 2011). Mousavi et al. indicated that the major increase of lactic acid in fermentation of pomegranate juice was observed in the log phase of the bacterial growth. *L. plantarum* produced significantly higher amount of lactic acid than that produced by *L. acidophilus* and *L. paracasei,* which is in good agreement with results of present study.

As the main product of LAB fermentation is lactic acid, according to Table 2, all parameters and their interactions had significant effect on production of lactic acid (*p* < .01). *Lactobacillus plantarum* and 1:1 mixture of *L. plantarum* and *L. casei* produced more lactic acid comparing to *L. casei* (*p* ≤.05). Other researchers reported some strains of *L. plantarum* are able to survive or even grow under the harsh conditions of the environment with high ethanol, low pH, and the presence of sulfite (Mendes Ferreira & Mendes‐Faia, 2020). Despite the good alcohol tolerance, that strain is homofermentative, for hexoses metabolism, which makes its application with no risk of volatile acid production (Krieger‐Weber et al., 2020).

More lactic acid was produced in media 2 compared to media 1 and 48 hr fermentation caused more lactic acid production comparing to 24 hr fermentation. Higher initial pH and presence of rice flour solution containing high amount of vitamin B and some Ca^2+^ in media was suitable for lactic acid fermentation. One cup of rice contains 5 mg Ca^2+^. Mortera et al. (Mortera et al., 2013) indicated that adding Ca^2+^ to media that contains citrate causes more consumption of citrate and consequently more production of lactic acid.

The three‐way interaction of selected lactic acid bacteria, media, and time on production of lactic acid indicated that fermentation of media 2 by *L. plantarum* and *L. casei* after 48 hr had greatest impact on lactic acid level. Third maximum level of lactic acid was observed in media 2 fermented by 1:1 mixture of *L. plantarum* and *L. casei* after 48 hr.

## 4 CONCLUSION

Overall, fermentation of red grape juice (media 1) and mixture of red grape juice and rice flour solution (media 2) by selected strains of lactic acid bacteria promoted considerable changes in the medium such as acid production, sugar consumption, and decreasing the pH as a result of bacterial growth during 48 hr fermentation. *Lactobacillus casei* versus *L. plantarum* showed less ability of fermentation in media 1, due to the low initial pH. In contrast, *L. Casei* showed good fermentation ability in media 2 because of the higher initial pH and adding rice flour solution (containing high amount of vitamin B). Fermentation by the mixture of *L. plantarum* and L. *casei* was more effective to increase microbial population; especially it was more impressive in media 2. Lactic acid was the main metabolite being produced during the fermentation by bacterial strains. Herein, it was demonstrated that the use of the mixture of red grape juice and rice flour solution be a proper substrate for producing lactic acid. Thus, it was revealed that, GRLp and GRLc produced the highest amount of lactic acid. Also, GRLpLc and GLp consumed higher amount of citric acid. All bacterial strains metabolized glucose and fructose with higher priority of glucose. Among all treatments, GRLpLc and GLpLc had higher affinity for glucose consumption. The outcomes of this study revealed that the mixture of red grape juice and rice flour solution is a suitable substrate for lactic acid fermentation.

## CONFLICT OF INTEREST

Authors declare that there are no conflict of interests.

## Data Availability

The authors confirm that the data supporting the findings of this study are available within the article. Raw data were generated at laboratory of Islamic Azad University. Derived data supporting the findings of this study are available from the corresponding author on request.
